# The effect of ginger supplementation on serum C-reactive protein, lipid profile and glycaemia: a systematic review and meta-analysis

**DOI:** 10.3402/fnr.v60.32613

**Published:** 2016-11-01

**Authors:** Mohsen Mazidi, Hong-Kai Gao, Peyman Rezaie, Gordon A. Ferns

**Affiliations:** 1 Key State Laboratory of Molecular Developmental Biology, Institute of Genetics and Developmental Biology, Chinese Academy of Sciences, Beijing, China; 2 Institute of Genetics and Developmental Biology, International College, University of Chinese Academy of Science, Beijing, China; 3 Department of General Surgery, The General Hospital of Chinese People’s Armed Police Forces, Beijing, China; 4 Biochemistry of Nutrition Research Center, School of Medicine, Mashhad University of Medical Science, Mashhad, Iran; 5 Division of Medical Education, Brighton and Sussex Medical School, University of Brighton, Brighton, United Kingdom

**Keywords:** meta-analysis, ginger, supplementation, C-reactive protein, fasting blood glucose, lipids

## Abstract

**Aim:**

To undertake a systematic review and meta-analysis of prospective studies to determine the effect of ginger supplementation on serum C-reactive protein (CRP), lipid profile, and glycaemia.

**Method:**

PubMed-MEDLINE, Web of Science, Cochrane Database, and Google Scholar databases were searched (up until July 2016) to identify prospective studies evaluating the impact of ginger supplementation on serum CRP. Random-effects model meta-analysis was used for quantitative data synthesis. Sensitivity analysis was conducted using the leave-one-out method. Heterogeneity was quantitatively assessed using the *I*^2^ index. Systematic review registration: CRD42016035973.

**Results:**

From a total of 265 entries identified via searches, 9 studies were included in the final selection. The meta-analysis indicated a significant reduction in serum CRP concentrations following ginger supplementation [weighted mean difference (WMD)−0.84 mg/L (95% CI −1.38 to −0.31, *I*^2^ 56.3%)]. The WMD for fasting blood glucose and HbA1c was −1.35 mg/dl (95% CI −2.04 to −0.58, *I*^2^ 12.1%) and −1.01 (95% CI −1.28 to −0.72, *I*^2^ 9.4%), respectively. Moreover, high-density lipoprotein and triglyceride significantly improved after ginger administration [1.16 mg/dl (95% CI 0.52 to 1.08, *I*^2^ 12.3%) and −1.63 mg/dl (95% CI −3.10 to −0.17, *I*^2^ 8.1%), respectively]. These findings were robust in sensitivity analyses. Random-effects meta-regression revealed that changes in serum CRP levels were independent of the dosage of ginger supplementation (slope −0.20; 95% CI −0.95 to 0.55; *p*=0.60).

**Conclusions:**

This meta-analysis suggests that ginger supplementation significantly reduces serum CRP and improves glycaemia indexes and lipid profile. Randomized control trials with larger sample size and with a longer-term follow-up period should be considered for future investigations.

Chronic inflammation has been associated with a wide range of diseases including cardiovascular disease (CVD), diabetes, arthritis, Alzheimer’s disease, pulmonary diseases, and autoimmune diseases (1, 2). C-reactive protein (CRP) is a plasma protein that rises in the systemic response to inflammatory conditions (3); in addition, there is a link between elevated level of this protein and major cardiovascular events (4, 5). Interleukin-6 (IL-6) is an important inflammatory cytokine that induces the hepatic production of CRP. A low-grade inflammation is a common feature of type 2 diabetes mellitus (DM2) and also has a role in the pathogenesis of its secondary complications such as atherothrombosis (6, 7). Inflammation also modifies insulin sensitivity, diabetes-related dyslipidaemia, and endothelial function (6, 8).

The rhizome of ginger (*Zingiber officinale* Roscoe, Zingiberaceae) is a widely used spice. For centuries, this plant plays a significant role in Chinese, Ayurvedic, and Unani-Tibb herbal medicine to treat cataract, rheumatism, nervous diseases, gingivitis, toothache, asthma, stroke, constipation, and diabetes (6, 9). Ginger contains active phenolic compounds such as gingerol, paradol, and shogaol that have antioxidant, anti-cancer, anti-inflammatory, and anti-atherosclerotic properties (1, 10).

Mechanisms of action include modulation of leukotriene (LT) and prostaglandin (PG) synthesis and inhibition of nuclear factor-κB (11). *In vitro*, the main components of ginger (gingerols and shogaols) can inhibit the synthesis of several pro-inflammatory cytokines including IL-1, tumour necrosis factor (TNF)-α, and IL-8 as well as PG and LT synthesis enzymes (6, 12). Mahluji et al. have previously shown that 2 g powdered ginger reduced plasma insulin, insulin resistance assessed by homeostatic model assessment (HOMA), serum fasting triglyceride (TG), and low-density lipoprotein (LDL), in type 2 diabetic patients; however, no significant changes were seen in blood glucose, total cholesterol, or high-density lipoprotein (HDL) levels (6). Bordia et al. have reported that ginger supplementation had no significant effect on blood glucose and serum lipids (8, 13).

Regarding the effect of ginger supplementation on CRP, Naderi et al. have reported that the concentration of inflammatory markers including CRP was reduced in the group treated with ginger compared with the group receiving placebo (5); moreover, Karimi et al. reported that the ginger supplementation caused a reduction of hs-CRP, IL-10, blood glucose, LDL, and TG, and an increase in HDL (14). Imani et al. indicated that daily administration of 1,000 mg ginger reduces serum fasting glucose (15). However, a few studies have reported an increase (16) or a non-significant (15) effect of ginger supplementation on inflammatory markers. Thus, inconsistent findings have been reported in this field.

Single studies to date have been limited by sample size, research design, and subject traits (gender, ethnicity, age, etc.) and underpowered to achieve a comprehensive and reliable conclusion. Meta-analysis has the benefit to overcome this limitation by increasing the sample size. Hence, the present study aimed to resolve this uncertainty by systematically reviewing the literature, and meta-analysis and meta-regression of all trials investigating the effects of ginger on serum CRP, blood lipids, and glycaemia.

## Materials and methods

### Literature search strategy

The present study was conducted according to the Preferred Reporting Items for Systematic Reviews and Meta-Analyses (PRISMA) Guidelines (17, 18). Moreover, the study protocol was registered with the International Prospective Register of Systematic Reviews, PROSPERO (registration no.: CRD42016038155). The primary exposure of interest was ginger administration while the primary outcome of interest was changes in CRP levels, lipid profile, and glycaemia subsequent to ginger administration. We searched multiple databases including PubMed-MEDLINE, Cochrane Central Register of Controlled Trials (CCTR), Cochrane Database of Systematic Reviews (CDSR), Web of Science; until July 2016 using a combination of search terms available in Supplementary Table 1. This was complemented by a physical search of the reference list of eligible articles and email correspondences with authors for additional data where relevant.

### Selection criteria

**Fig. 1:**
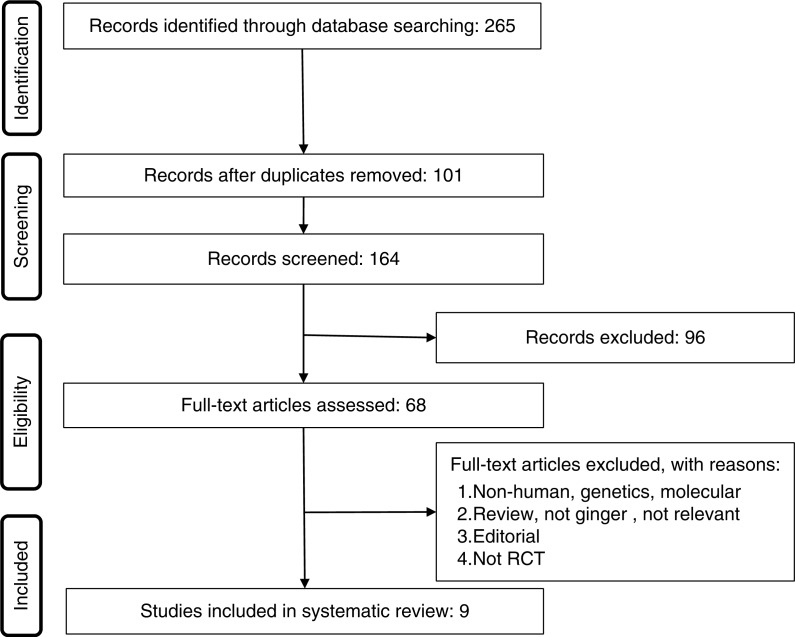
PRISMA flow chart for the studies selection.

We included all randomized control trials (RCTs) evaluating the effect of ginger administration on the outcomes of interest. Eligible studies had to meet the following criteria: 1) being a controlled trial with either parallel or crossover design, and 2) presentation of sufficient information on primary outcome at baseline and at the end of follow-up in each group or providing the net change values. Exclusion criteria were: 1) non-clinical studies; 2) observational studies with case-control, cross-sectional or cohort design; and 3) studies that did not provide mean (or median) plasma concentrations of our interested outcomes at baseline and/or at the end of trial. Narrative reviews, comments, opinion pieces, methodological, editorials, letters, or any other publications lacking primary data and/or explicit method descriptions were also excluded. Study selection started with the removal of duplicates, followed by titles and abstracts screening by two reviewers. To avoid bias, they were blinded to the names, qualifications, or the institutional affiliations of the study authors. The agreement between the reviewers was excellent (κ index: 0.89; *p<*0.001). Disagreements were resolved at a meeting between reviewers prior to selected articles being retrieved (Fig. 1).

### Data extraction and management

The full text of studies meeting inclusion criteria was retrieved and screened to determine eligibility by two reviewers (MM and PR). Following assessment of methodological quality, the two reviewers extracted data using a purpose-designed data extraction form and independently summarized what they considered to be the most important results from each study. These summaries were compared and any differences of opinion were resolved by discussion and consultation with a third reviewer. Any further calculations on study data considered necessary were conducted by the first reviewer and checked by the second reviewer. Descriptive data extracted included the first author, reference, country, study design, inclusion criteria, treatment duration, sample size, study groups, age (years), female (*n*,%), and ginger dose.

### Quality assessment

A systematic assessment of bias in the included RCTs was performed using the Cochrane criteria (19). The items used for the assessment of each study were the following: adequacy of random sequence generation, allocation concealment, blinding of participants, personnel and outcome assessment, handling of drop-outs (incomplete outcome data), selective outcome reporting, and other potential sources of bias. According to the recommendations of the Cochrane Handbook, a judgment of ‘yes’ indicated low risk of bias, while ‘no’ indicated high risk of bias. Labelling an item as ‘unclear’ indicated an unclear or unknown risk of bias.

### Data synthesis

Based on recommendation within the Cochrane Handbook, the mean change from baseline in interested variable concentrations and standard deviation (SD) for both intervention and control groups was used to calculate the effect size (19). In brief, net changes in measurements (change scores) were calculated as follows: measure at end of follow-up - measure at baseline (20). Where standard error of the mean (SEM) was only reported, SD was estimated using the following formula: SD=SEM×square root (*n*), where *n* is the number of subjects (20). If the outcome measures were reported as median and range (or 95% confidence interval (CI)], mean and standard SD values were estimated using the method described by Hozo et al. (21). When the outcome variable was available only in the graphic form, the software GetData Graph Digitizer 2.24 (20) was used to digitize and extract the data. Blood lipid and glucose levels were collated in mmol/L; a multiplication factor of 0.0259, 0.0113, or 0.0555 was used to convert cholesterol (total cholesterol, HDL-C, or LDL-C), TGs, and glucose levels, respectively, from mg/dl to mmol/L as appropriate (20).

A random-effects model (using the DerSimonian–Laird method) and the generic inverse variance method were used (22). Heterogeneity was quantitatively assessed using *I*^2^ index. *I*^2^ values <50% and ≥50% corresponded with the use of fixed-effects and random-effects model, respectively (20). Effect sizes were expressed as weighed mean difference (WMD) and 95% CI. In order to evaluate the influence of each study on the overall effect size, a sensitivity analysis was conducted using the leave-one-out method (i.e. removing one study each time and repeating the analysis) (23–25).

### Meta-regression

Random-effects meta-regression was performed using the unrestricted maximum likelihood method to evaluate the association between calculated WMD and potential moderator including dose of ginger administration.

### Publication bias

Potential publication bias was explored using a visual inspection of Begg’s funnel plot asymmetry, Begg’s rank correlation, and Egger’s weighted regression tests (20). Duval and Tweedie’s ‘trim and fill’ and ‘fail-safe N’ methods were used to adjust the analysis for the effects of publication bias (26). Meta-analysis was conducted using comprehensive meta-analysis (CMA) V3 software (Biostat, NJ) (27).

## Results

### Summary of searches and study selection process

A total of 145 unique citations were identified from searches, of which 99 records remained after removing duplicates. After screening via titles and abstracts, 21 articles remained for further evaluation, of which 16 were excluded for the following reasons: non-human studies, genetic, or molecular studies (*n=*4); reviews or editorial articles (*n=*7); and not RCTs (*n=*3), short follow-up duration (*n=*2) (Fig. 1). Therefore, nine studies were included in the final meta-analysis.

### Risk of bias assessment

There was a lack of information about blinding of participants; however, all the evaluated studies had a low risk of bias according to selective outcome reporting. Details of the quality of bias assessment are shown in Supplementary Table 2.

### Characteristics of the included studies

The characteristics of the included studies are summarized in Table 1. These studies were published between 2008 and 2015 from Iran (eight studies) and the United States of America (one study). The number of participants included in these studies ranged from 10 (28) to 88 (29). The mean age of participants ranged from 23.7 years (30) to 58 years (31). A range of doses from 1 to 3 g per day was administered in these trials. Duration of ginger supplementation ranged from 8 weeks to 3 months. Among the nine studies included in the meta-analysis, four articles included patients with type 2 diabetes (8, 29, 32, 33), two articles included patients undergoing peritoneal dialysis (PD) (15, 31), one article included obese patients (BMI ≥ 30 kg/m^2^) (30), one article included patients with BMI 25–29.9 kg/m^2^ (28), and one article included patients with hyperlipidaemia (34). Ginger appeared safe and well-tolerated in all RCTs included in this analysis, with no reports of any serious adverse events. Demographic and baseline parameters of the included studies are shown in Table 1.

**Table 1:** General characteristics of nine studies eligible for inclusion in meta-analysis

First author, reference #	Country	Study design	Inclusion criteria	Treatment duration	Sample size	Age (years)	Female (*n*,%)	Ginger dose
Arablou T. 2014 (8)	Iran	Double-blinded, placebo-controlled clinical trial	Patients 30–70 years old with type 2 diabetes	12 weeks	63	Ginger group (52.6±8.4) Placebo group (52.0±9.0)	Ginger group (75.8%) Placebo group (76.7%)	1,600 mg/day
Atashak S. 2010 (30)	Iran	Randomized double-blind, placebo-controlled trial	Obese men (BMI ≥30 kg/m^2^, aged 18–30 years)	10 weeks	32	Ginger group (23.7) Placebo group (25.4)	(0%)	1 g/day
Imani H. 2015 (15)	Iran	Randomized, double-blind, placebo-controlled trial	Patients undergoing continuous ambulatory peritoneal dialysis in the age range of 29–79 years	10 weeks	36	Ginger group (56±2.5) Placebo group (58±3)	Ginger group (39%) Placebo group (44%)	1,000 mg/day
Mansour M. 2012 (28)	USA	Randomized crossover study	Men, age 19–50 years, BMI 25–29.9 kg/m^2^	Not mentioned	10	39.1±3.3	0	2 g/day
Shidfar F. 2015 (33)	Iran	Double-blind, placebo-controlled, randomized clinical trial	20- to 60-year-old patients with type 2 diabetes who did not receive insulin	3 months	45	Ginger group (45.2±7.64) Placebo group (47.1±8.31)	Fill in	3 g/day
Alizadeh-Navaei R. 2008 (34)	Iran	Double-blind controlled clinical trial study	Patients with hyperlipidaemia	45 days	85	Ginger group (53.8±11.8) Placebo group (53.5±11)	Ginger group (64.4%) Placebo group (55%)	3 g/day
Mahluji S. 2013 (32)	Iran	Randomized, double-blind, placebo-controlled trial	Patients with type 2 diabetes	2 months	54	Ginger group (49.2±5.1) Placebo group (53.1±7.9)	Ginger group (46%) Placebo group (42%)	2 g/day
Mozaffari-Khosravi H. 2014 (29)	Iran	Randomized, double-blind, placebo-controlled trial	Type 2 diabetes	2 months	88	Ginger group (49.83±7.23) Placebo group (51.05±7.70)	Ginger group (56.1%) Placebo group (67.5%)	3 g/day
Tabibi H. 2016 (31)	Iran	Randomized, double-blind, placebo-controlled trial	Peritoneal dialysis	10 weeks	36	Ginger group (56.0±2.5) Placebo group (58.0±3.0)	Ginger group (39%) Placebo group (44%)	1 g/day

### Pooled estimate of the effect of ginger administration on CRP

**Fig. 2:**
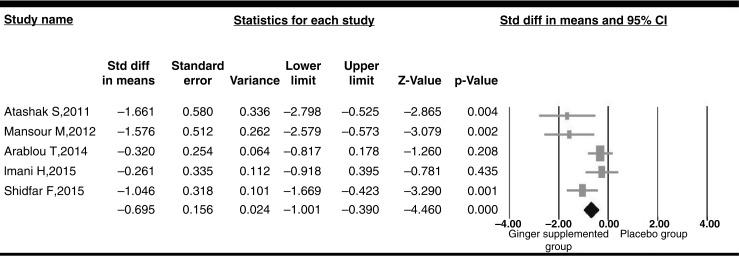
The pooled estimate (weighted mean difference) of the effect of ginger administration on CRP levels.

The pooled estimate (WMD) of the effect of ginger administration on CRP levels was −0.84 mg/L (95% CI−1.38 to −0.31, heterogeneity *p=*0.053) across all studies (Fig. 2). The pooled estimate (WMD) of the effect of ginger administration on fasting blood glucose (FBG) levels was −1.35 mg/dl (95% CI−2.04 to −0.58, heterogeneity *p=*0.056) across all studies. Pooled estimate of the effect of ginger on lipid profile and HbA1c is reported in Table 2.

**Table 2:** The pooled estimate (weighted mean difference) of the effect of ginger administration on glycaemia and lipid profile

Variables	Result of meta-analysis
Low-density lipoprotein	−1.33 mg/dl (95% CI −2.54 to −0.11)
High-density lipoprotein	1.16 mg/dl (95% CI 0.52 to 1.08)
Total cholesterol	−0.22 mg/dl (95% CI −0.06 to 0.48)
Triglyceride	−1.63 mg/dl (95% CI −3.10 to −0.17)
HbA1c	−1.01% (95% CI −1.28 to −0.72)

### Sensitivity analysis

In leave-one-out sensitivity analyses, the pooled effect estimates remained similar for both CRP and FBG: −0.84 mg/l (95% CI −1.38 to −0.31) and−1.75 mg/dl (95% CI −2.66 to −0.84), respectively. This result confirms that the significant difference between the studied groups is the overall effect of all included studies.

### Meta-regression

**Fig. 3:**
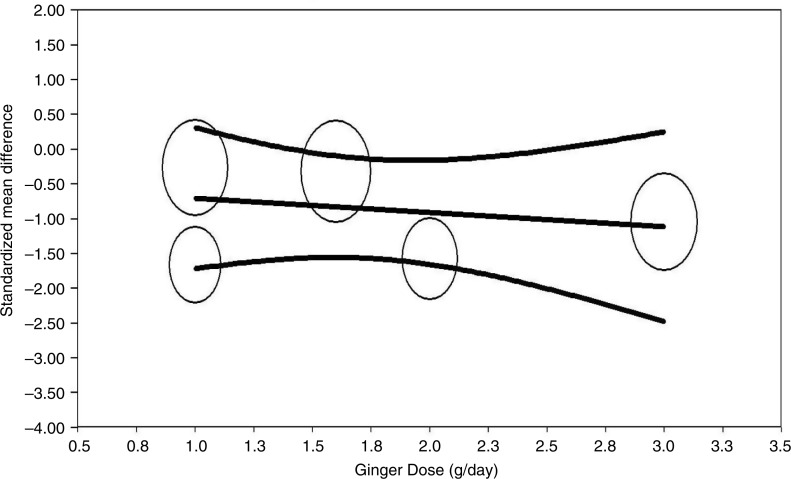
Regression of standardized mean difference on dose. Meta-regression plots of the association between mean changes in C-reactive protein (CRP) after ginger supplementation with dose of treatment. Circles represent each study, middle line is regression line, and two lines around the middle line represent the 95% confidence interval.

Random-effects meta-regression was performed to evaluate the impact of potential moderators on the estimated effect size. Changes in plasma CRP levels were independent of the dosage of ginger administration (slope −0.20; 95% CI −0.95 to 0.55; *p*=0.60; Fig. 3).

### Publication bias

**Fig. 4:**
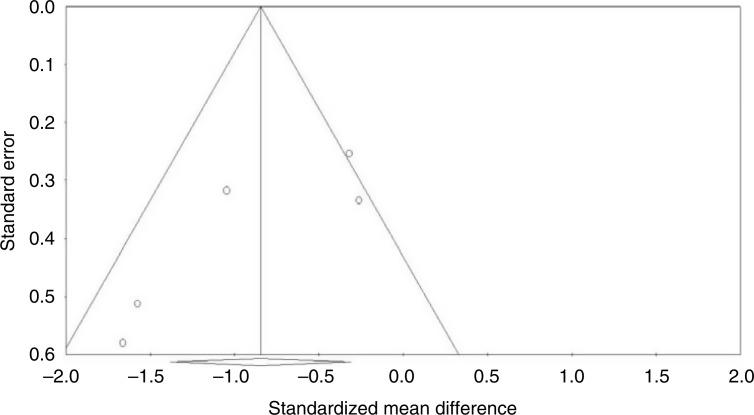
Funnel plots detailing publication bias in the studies selected for analysis. Open circles represent observed published studies; open diamond represents observed effect size.

**Fig. 5:**
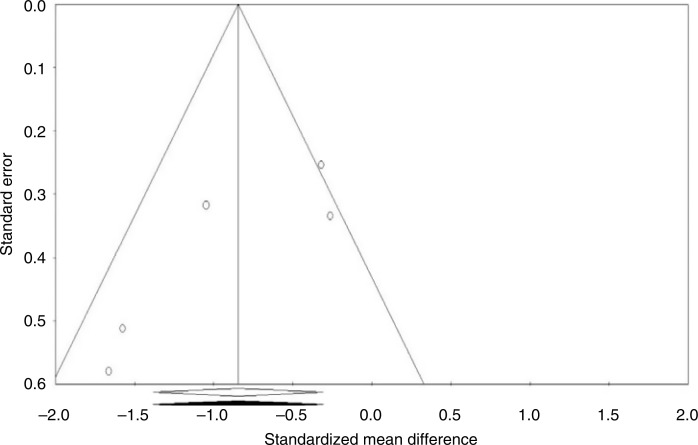
Trim and fill method was used to impute for potentially missing studies, no potentially missing study was imputed in funnel plot. Open circles represent observed published studies; open diamond represents observed effect size; closed diamond represents imputed effect size.

Visual inspection of funnel plot symmetry suggested no potential publication bias for the comparison of plasma CRP levels between ginger-administrated groups and placebo groups (Fig. 4). Moreover, the Egger’s linear regression (intercept=−4.25, standard error=1.7; 95% CI −9.6 to 1.1, *t*=2.48, df=3.00, two-tailed *p=*0.088) and Begg’s rank correlation test (Kendall’s τ with continuity correction=−0.600, *z*=1.46, two-tailed *p=*0.14) were not indicative for publication bias. After adjustment of effect size for potential publication bias using the ‘trim and fill’ correction, no potentially missing study was imputed in the funnel plot (WMD −0.84 mg/L, 95% CI −1.38 to −0.31; Fig. 5). The ‘fail-safe N’ test showed that 120 studies would be needed to bring the WMD down to a non-significant (*p*>0.05) value.

## Discussion

This meta-analysis suggests that ginger administration significantly reduced CRP level and improved glycaemia index and lipid profile. In agreement with our findings, some of the included studies have reported that ginger (*Z. officinale*) reduces inflammatory markers (8, 32). Arablou et al. indicated that consumption of ginger powder for 12 weeks can reduce CRP significantly in patients with type 2 diabetes (8). Their findings are in line with the result of Atashak et al. (31), which showed that consumption of 1 g of powdered ginger daily for 10 weeks led to a 27.6% reduction in mean CRP levels in obese men. Imani et al. have reported that daily administration of 1,000 mg ginger had no effect on serum CRP in patients on PD and stated that the reason for this disparity may be due to the administration of a higher dose of ginger in the other studies (15).

It should be noted that there are contradictory findings about the effects of ginger supplementation on inflammatory marker between studies, which are not included in this meta-analysis. Naderi et al. conducted a 12-week clinical trial to investigate the effects of ginger supplementation on nitric oxide and CRP in elderly knee osteoarthritis patients and reported that ginger powder supplementation at a dose of 1 g/d can reduce inflammatory markers in patients with knee osteoarthritis (5), which is in line with the findings of a study by Rahimlou et al. (35). However, one study reported that after oral administration of 100–1,000 mg/ml squeezed ginger extract in mice, the production of inflammatory markers increased (16).

Chronic inflammation and activation of the innate immune system are strongly involved in the pathogenesis of diabetes (8, 36). In addition, it has been stated that the inflammatory marker CRP in adults has value for treatment initiation in individuals with intermediate CVD risk (38). Regarding the mechanism of the effect of ginger on PGE2, an inhibition of cyclooxygenase-2 mRNA expression and direct inhibition of this enzyme activity is proposed (8, 38). Furthermore, it has been reported that the effect of ginger on inflammation is also due to the effect of certain active compounds (gingerols and zerumbone) that inhibit NF-kB and TNF-α expression in liver cancer cells (1). 6-Gingerol and 6-paradol have strong and effective anti-inflammatory activity and suppress TNF-α production (8, 39). This inhibition decreases NF-κB activity in addition to other inflammatory cytokines as well as cyclooxygenase 2 and its associated products including PGE2. Therefore, acute-phase proteins such as CRP are also inhibited in this process.

Moreover, other possible mechanisms are proposed regarding pharmacological activity of ginger. Ginger suppresses LT biosynthesis by inhibiting 5-lipoxygenase (6, 40) l, and ginger extract was found to inhibit β-amyloid peptide-induced cytokine and chemokine expression in a cell line of human monocytes (6, 41).

Several papers have proposed that the hypoglycaemic and other pharmacological activities of ginger are due to its content of phenols, polyphenols, and flavonoids (42). *In vitro* studies on the mechanism of the effect of ginger on glucose metabolism have shown that the active constituents of ginger including 6-gingerol and 8-gingerol enhanced cellular glucose uptake by increasing gene expression of glucose transporter type 4 (43, 44). Another proposed mechanism is that ginger decreases blood glucose by antagonistic activity against serotonin receptors (8, 45). Moreover, several studies have reported that ginger supplementation can affect glucose transport and tolerance in type 2 diabetic patients with insulin resistance. Isa et al. indicated that the 6-gingerol and 6-shogaol in ginger upregulate adiponectin, and 6-shogaol has agonistic activity with PPARγ. Thus, increasing adiponectin improves insulin sensitivity (46, 47).

We acknowledge several limitations in our review and meta-analysis. First, as with any meta-analysis, internal validity relies on the quality of individual studies. Several limitations can be named in this regard. Most of the included studies had relatively medium sample sizes, potentially leading to overestimation of treatment effects; smaller trials might be methodologically less robust and more prone to report larger effect sizes (48, 49). The number of available studies concerning the described topic was rather small. Moreover, most of the studies were conducted in clinical populations rather than in generally healthy populations, and this is likely to affect the baseline levels of CRP and the inflammatory markers.

## Conclusion

This systematic review showed that ginger supplementation can improve CRP level, glycaemia indexes, and lipid profile, which can be useful for the prevention and management of CVD. RCTs with a larger sample size and a longer follow-up period should be considered for future investigations to give an unequivocal answer as to whether ginger can reduce CRP and improve glycaemia indexes and lipid profile.

## Supplementary Material

The effect of ginger supplementation on serum C-reactive protein, lipid profile and glycaemia: a systematic review and meta-analysis
